# Deep Learning Improves Osteonecrosis Prediction of Femoral Head After Internal Fixation Using Hybrid Patient and Radiograph Variables

**DOI:** 10.3389/fmed.2020.573522

**Published:** 2020-10-07

**Authors:** Wanbo Zhu, Xianzuo Zhang, Shiyuan Fang, Bing Wang, Chen Zhu

**Affiliations:** ^1^Department of Orthopedics, The First Affiliated Hospital of USTC, Division of Life Sciences and Medicine, University of Science and Technology of China, Hefei, China; ^2^Department of Orthopedics, Affiliated Anhui Provincial Hospital of Anhui Medical University, Hefei, China; ^3^School of Electrical and Information Engineering, Anhui University of Technology, Ma'anshan, China

**Keywords:** osteonecrosis, femoral neck fracture, clinical prediction, artificial intelligence, nomogram

## Abstract

Femoral neck fractures (FNFs) are a great public health problem that leads to a high incidence of death and dysfunction. Osteonecrosis of the femoral head (ONFH) after internal fixation of FNF is a frequently reported complication and a major cause for reoperation. Early intervention can prevent osteonecrosis aggravation at the preliminary stage. However, at present, failure to diagnose asymptomatic ONFH after FNF fixation hinders effective intervention at early stages. The primary objective of this study was to develop a predictive model for postoperative ONFH using deep learning (DL) methods developed using plain X-ray radiographs and hybrid patient variables. A two-center retrospective study of patients who underwent closed reduction and cannulated screw fixation was performed. We trained a convolutional neural network (CNN) model using postoperative pelvic radiographs and the output regressive radiograph variables. A less experienced orthopedic doctor, and an experienced orthopedic doctor also evaluated and diagnosed the patients using postoperative pelvic radiographs. Hybrid nomograms were developed based on patient and radiograph variables to determine predictive performance. A total of 238 patients, including 95 ONFH patients and 143 non-ONFH patients, were included. A CNN model was trained using postoperative radiographs and output radiograph variables. The accuracy of the validation set was 0.873 for the CNN model, and the algorithm achieved an area under the curve (AUC) value of 0.912 for the prediction. The diagnostic and predictive ability of the algorithm was superior to that of the two doctors, based on the postoperative X-rays. The addition of DL-based radiograph variables to the clinical nomogram improved predictive performance, resulting in an AUC of 0.948 (95% CI, 0.920–0.976) and better calibration. The decision curve analysis showed that adding the DL increased the clinical usefulness of the nomogram compared with a clinical approach alone. In conclusion, we constructed a DL facilitated nomogram that incorporated a hybrid of radiograph and patient variables, which can be used to improve the prediction of preoperative osteonecrosis of the femoral head after internal fixation.

## Introduction

Hip fracture is a significant public health concern that affects 4.5 million people worldwide each year and this number is expected to increase to 21 million in the next 40 years (1, 2). Femoral neck fracture (FNF) is one of the most common types of hip fracture, accounting for 49–80% of all hip fractures (3, 4). Despite the availability of multiple effective internal fixation procedures, ~10–48.8% femoral neck fractures require reoperation (5–7). Osteonecrosis of the femoral head (ONFH) is a major cause of reoperation for FNF (8). Joint disfunction, pain, disability, and mental anguish caused by ONFH result in great suffering for patients (9–11). End-stage ONFH often inevitably requires artificial joint replacement surgery, an invasive and economically costly technique. Early diagnosis can facilitate the application of interventions that can avoid or delay arthroplasty to a certain extent (12–14). However, misdiagnoses and delayed diagnoses are common due to the lack of preliminary symptoms, typical features, and internal fixation interference on radiographs (14). Different diagnostic criteria or simple visual estimates are used by radiologists for practical imaging diagnosis, resulting in unsatisfactory levels of diagnostic consistency and accuracy (15). Therefore, early accurate and consistent prediction of ONFH in patients after FNF internal fixation may hold the key for improving patient outcomes.

Deep learning (DL) using radiographs has a proven ability of classifying bone structures and features in specific sites with expert-level accuracy (16, 17). Convolutional Neural Networks (CNNs) are the most suitable models for image recognition of DL, and have been widely used for the orthopedic diagnosis of wrists and ankles (18, 19). Gale et al. developed a hip fracture detector using DL and achieved an AUC of 0.994 (20). Cheng et al. reported on a deep convolutional neural network (DCNN) for the detection and localization of hip fractures using pelvic radiographs, which achieved an AUC of 0.98 for the identification of hip fractures (21). Recently, Chee et al. made a breakthrough discovery for the diagnosis of early ONFH using radiography through deep learning (22). This model achieved an AUC of 0.93 and sensitivity and specificity that were not inferior to the diagnosis made by both the less experienced and experienced radiologists. Their study indicated the potential of DL for the diagnosis and prediction of ONFH, especially for X-ray imaging. However, the implementation of DL for the diagnosis of postoperative ONFH using digital radiography remains unexplored. Postoperative X-rays are highly affected by interference, such as that of internal fixation devices, which cause difference between the images on radiographs and the original appearance of the femoral neck and femoral head. Since postoperative X-rays are the most common method used for early examination, a consistent diagnosis based on postoperative X-rays made using DL may improve the prediction of postoperative ONFH for better prognosis. In this study, we designed and assessed the diagnostic performance of a DL algorithm based on the CNN network model using postoperative X-rays. We also compared the accuracy of the diagnosis of postoperative ONFH between this DL model and assessments made by two orthopedic doctors of different levels of experience.

In previous studies, a large number of research studies have indicated that patient and interventional variables, including demography, fracture classification, laboratory examination, reduction quality, and initial postoperative rehabilitation, are significantly associated with postoperative ONFH (23–26). However, intraoperative, and postoperative factors, especially radiographic variables, including intraoperative reduction and fracture healing, have yet to be incorporated into routine clinical postoperative ONFH prediction. In this study, a DL facilitated predictive model using a hybrid of patient and artificial intelligence (AI) radiographic variables, was also developed. Comparisons were made with a single clinical prediction model was performed to estimate whether DL could improve the prediction of postoperative ONFH.

## Materials and Methods

### Study Population

Data were obtained from two urban tertiary hospitals, The First Affiliated Hospital of University of Science and Technology of China (FAH) and the Southern Branch of the First Affiliated Hospital of University of Science and Technology of China (SBH). One hundred thirty-nine FAH patients and 99 SBH patients who had received closed reduction and cannulated screw fixation from June 2013 to January 2015 were enrolled in this study. The patient inclusion criteria were as follows: (i) Patients over 18 years of age with fresh FNFs; (ii) Postoperative pelvic radiographs obtained 6 months after surgery; (iii) Continuous follow-up for a minimum of 5 years with the clinical characteristics available. The exclusion criteria were as follows: (i) Pathological fractures and bilateral fractures; (ii) Long-term hormone use. The treatment standard and strategy used for femoral neck fracture was the cannulated compression screws fixation technique, based on American Academy of Orthopedic Surgeons guidelines (27). Postoperative ONFH was diagnosed using pelvic MRIs or co-diagnosis by three experienced orthopedic surgeons based on the pelvic radiograph obtained at the last follow-up. This study was approved by the Ethics Committees of both hospitals. Exemption of the informed consent, the information disclosure, and a negative opportunity are guaranteed in the Ethical approval (20-P-049).

Demographics, comorbidities, smoking status, alcohol use, blood tests, preoperative Garden classification, Pauwels angle, preoperative interval from injury, operation associated data, postoperative Garden index, preoperative interval to weight bearing and other baseline patient and clinical data were derived from medical and follow-up records. The data were de-identified after patient variables were collected.

### Imaging Studies

Image acquisition and retrieval procedures were conducted using Picture Archiving and Communication Systems (PACS) on FAH and SBH patients. Digital radiographs of the hip were obtained using Digital Diagnostics (Philips Healthcare) on FAH patients and Discovery XR656 (GE Healthcare) on SBH patients. The size of the stored images varied from 2,128 × 2,248 pixels to 2,688 × 2,688 pixels, with 8-bit grayscale color. Each radiograph was labeled based on the final diagnosis of postoperative ONFH. Geometric, smooth, concave, bandlike low-signal intensity lesions at the femoral head on the T1-weighted images were regarded as pathognomonic MRI findings of ONFH. For MRI data not obtained at the last follow-up (45/238, 18.9%), diagnosis was based on pelvic plain radiographs obtained at the last follow-up and was set as a reference for labeling. The Association Research Circulation Osseous (ARCO) classification system was used as the diagnostic standard for ONFH (28).

Radiographic image files were loaded for processing using a MATLAB library (version 2017b, MathWorks, USA). The 7 × 7 cm images centered on the bilateral femoral heads were cropped. The center coordinates were manually recorded in advance. Radiographs were standardized to a common size and pixel intensity distribution. The images were down-sampled and padded to a final size of 120 × 120 pixels. Mean pixel intensity and standard deviation of each image was normalized.

### Algorithm Development and Extraction of Image Variables

For the development of a deep learning algorithm, we used MATLAB (version 2017b, MathWorks, USA) to implement a CNN model to compute abstract image features from input image pixel arrays. The design of the CNN model is shown in Table 1. The CNN model consisted of three convolutional blocks, a dropout and full connection layers. Each convolutional block comprised of convolutional operation, batch normalization, relu, and average pooling. The input used was Pixel values were set at 120^*^120 using a digital image. Cubic convolution and pooling were performed on each layer to adjust the weights of the neural network, using the difference between the output and true labels.

**Table 1:** The design of CNN model.

**Type**	**Operations**	**Filter shape**	**Input size**
Conv1	Conv	8 × 7 × 7 × 1	120*120
	batchnorm		
	relu		
	avgpool		8 × 120*120
Conv2	Conv	7 × 5 × 5 × 8	8 × 60*60
	batchnorm		
	relu		
	avgpool		7 × 60*60
Conv3	Conv	5 × 3 × 3 × 7	7 × 30*30
	batchnorm		
	relu		
	avgpool		5 × 30*30
Dropout	Dropout	1*1	5 × 15*15
FC	Fully connected	1,125*1	5 × 15*15
Regression	Regression output	1*1	1*1

The patients in the dataset were assigned to different groups as follows: 149 (63%) for training, 17 (7%) for validation and 72 (30%) for testing. The output results underwent regression analysis. The network output was a probability distribution for the continuous variables of the regression coefficient from 0 to 1.25, which was divided at 0.25 intervals into classified labels, 1–5. Higher label values were more likely to be considered to more strongly predict postoperative ONFH. In this study, this output label was referred to as the AI index classification.

### Algorithm Evaluation

Seventy-two independent datasets were used to test the trained predictive model to evaluate its accuracy for postoperative ONFH prediction. The probability of the diagnosis being postoperative ONFH generated by the model was evaluated using the receiver operating characteristic (ROC) curve and the area under the curve (AUC). The sensitivity, accuracy, recall and specificity of the radiographs for the prediction of ONFH were measured using a cutoff level probability of 0.5. A training curve was used to determine root mean squared error (RMSE) and loss, while a precision-recall curve was used to determine precision and recall.

### Image Predictive Variable Evaluation

We compared the AI index with the predictive measurement scores assigned by the two orthopedic surgeons of different levels of experience with the results of the DL algorithm based on the same X-rays to evaluate the performance of the algorithm. Radiographs obtained 6-months after anteroposterior hip operations were randomly divided into two IPAC sequences by the study coordinator. A less experienced orthopedic doctor (Doctor A, 3rd year of residency in orthopedics) and an experienced orthopedic doctor (Doctor B, 18 years in orthopedics) participated in the reading session. Both doctors were not involved in surgery, data collection or reference labeling. A score based on the subjective prediction of the doctors using the postoperative X-ray to determine the most likely outcome at final follow-up was assigned using a 1–5 grading system. One indicated that the development of ONFH was considered to be impossible, while 5 indicated that the development of ONFH was considered to be certain. Each doctor independently graded the predictive variables for ONFH. Comparison between the performance of the AI index and the evaluation made by the two doctors was conducted through calibration and ROC analysis.

### Development of Prediction Models

**Figure 1:**
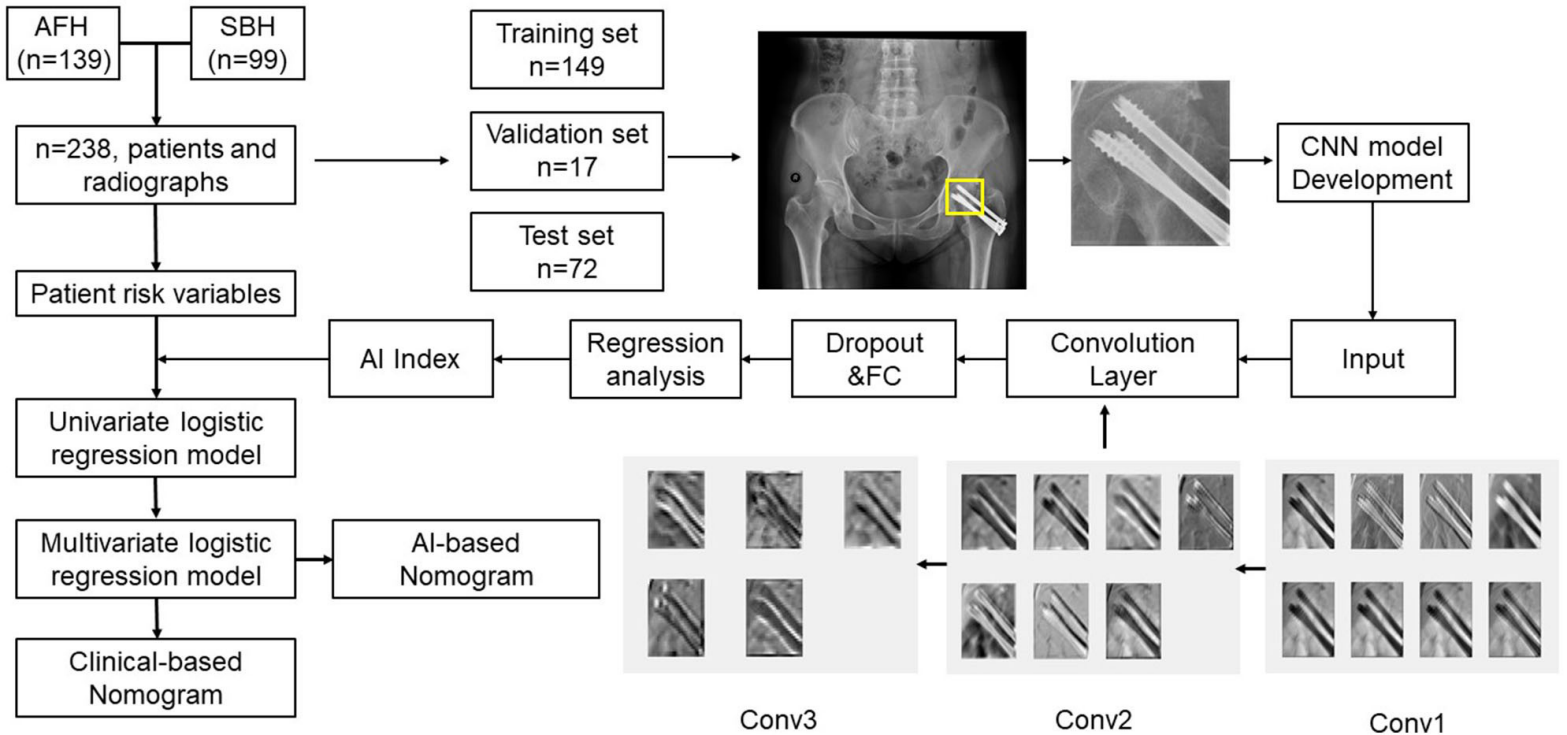
Flowchart of hybrid nomogram construction.

A multivariable logistic regression analysis was used to develop the clinical predication model based on patient and clinical variables. AI index classification was applied as a candidate predictor for univariate and multivariable logistic regression analyses for the construction of a DL-based postoperative ONFH prediction model using hybrid variables. A clinical prediction nomogram and a DL-based nomogram were then constructed based on multivariate logistic regression models. The work flowchart of this study is presented in Figure 1.

### Assessment of Nomogram Performance

AI-based nomogram and clinical nomogram calibration were assessed using a calibration curve. The discrimination performance of both the AI-based nomogram and clinical nomogram were quantified using the AUC.

### Clinical Use

Decision curve analysis (DCA) was performed by calculating the net benefits for a range of threshold probabilities to estimate the clinical utility of the nomogram.

### Statistical Analysis

Median and mean standard deviation (SD) were used to describe continuous variables. Categorical variables were presented as frequencies and percentages. Statistical comparisons between groups were performed using the Mann-Whitney *U*-test and Chi-square test. R software version 3.0.1 was used to construct the nomogram. The “pROC” package was used to plot ROC curves. Nomogram construction and calibration plot creation were performed using the “rms” package. DCA was performed using the “dca.R” package. Model selection was based on the forward–backward step-wise method using the likelihood ratio test with Akaike's information criterion as the stopping rule. The model with the smallest Akaike Information Criterion was selected as the final model. The statistical significance levels reported are all two-sided, with statistical significance set at a *P*-value of 0.05.

## Results

### Patient and Radiograph Characteristics

Postoperative radiographs of a total of 238 patients, including 95 ONFH patients and 143 normal patients were used for the development of the DL model and construction of the predictive nomogram. Imaging feature variables were extracted from each radiograph and were referred to as the AI index of all patients. Table 2 shows the baseline characteristics of the patients. Significant differences were found in BMI, Charlson comorbidity index, Injury Severity Score (ISS), d-dimer, timing of reduction, Garden classification and AI index between patients with ONFH and those without ONFH (Table 2).

**Table 2:** Patients baseline characteristics stratified by ONFH.

	**All patients**	**Non-ONFH group**	**ONFH group**	***p***
	***N = 238***	***N = 143***	***N = 95***	
Age	46.4 ± 12.7	45.6 ± 13.3	47.6 ± 11.7	0.215
Sex				0.167
Female	106 (44.5%)	58 (40.6%)	48 (50.5%)	
Male	132 (55.5%)	85 (59.4%)	47 (49.5%)	
BMI	22.7 ± 2.88	22.4 ± 2.82	23.2 ± 2.93	0.048
Smoking				0.875
No	148 (62.2%)	90 (62.9%)	58 (61.1%)	
Yes	90 (37.8%)	53 (37.1%)	37 (38.9%)	
Alcohol use				0.696
No	165 (69.3%)	101 (70.6%)	64 (67.4%)	
Yes	73 (30.7%)	42 (29.4%)	31 (32.6%)	
WIC	1.34 ± 1.40	1.10 ± 1.21	1.68 ± 1.59	0.003
CVD				0.097
No	220 (92.4%)	136 (95.1%)	84 (88.4%)	
Yes	18 (7.56%)	7 (4.90%)	11 (11.6%)	
ISS score				0.029
≤ 16	210 (88.2%)	132 (92.3%)	78 (82.1%)	
>16	28 (11.8%)	11 (7.69%)	17 (17.9%)	
WBC	7.42 ± 2.44	7.51 ± 2.51	7.28 ± 2.34	0.484
RBC	4.30 ± 0.57	4.31 ± 0.59	4.28 ± 0.55	0.638
Hb	130 ± 16.4	130 ± 16.5	131 ± 16.3	0.871
PLT	181 ± 58.2	178 ± 56.6	185 ± 60.6	0.387
ALB	40.9 ± 3.18	41.1 ± 3.18	40.7 ± 3.19	0.360
D-dimer	4.40 ± 5.59	5.16 ± 6.40	3.27 ± 3.86	0.005
Causes of injury				0.192
High energy trauma	63 (26.5%)	33 (23.1%)	30 (31.6%)	
Low energy trauma	175 (73.5%)	110 (76.9%)	65 (68.4%)	
Timing of reduction				<0.001
<72 h	100 (42.0%)	72 (50.3%)	28 (29.5%)	
72–120 h	97 (40.8%)	58 (40.6%)	39 (41.1%)	
>120 h	41 (17.2%)	13 (9.09%)	28 (29.5%)	
ASA grade				0.223
Grade 1	118 (49.6%)	76 (53.1%)	42 (44.2%)	
Grade 2–3	120 (50.4%)	67 (46.9%)	53 (55.8%)	
Garden classification				0.014
Type 2	19 (7.98%)	17 (11.9%)	2 (2.11%)	
Type 3	116 (48.7%)	63 (44.1%)	53 (55.8%)	
Type 4	103 (43.3%)	63 (44.1%)	40 (42.1%)	
Pauwels angle	53.2 ± 14.8	53.9 ± 15.4	52.1 ± 13.8	0.346
Garden index				0.130
1	43 (18.1%)	29 (20.3%)	14 (14.7%)	
2	61 (25.6%)	34 (23.8%)	27 (28.4%)	
3	70 (29.4%)	36 (25.2%)	34 (35.8%)	
4	64 (26.9%)	44 (30.8%)	20 (21.1%)	
Interval to part weightbearing				0.393
<1 m	16 (6.72%)	10 (6.99%)	6 (6.32%)	
1–3 m	89 (37.4%)	58 (40.6%)	31 (32.6%)	
3–6 m	122 (51.3%)	67 (46.9%)	55 (57.9%)	
>6 m	11 (4.62%)	8 (5.59%)	3 (3.16%)	
Interval to full weightbearing				0.474
<3 m	25 (10.5%)	15 (10.5%)	10 (10.5%)	
3–6 m	161 (67.6%)	93 (65.0%)	68 (71.6%)	
>6 m	52 (21.8%)	35 (24.5%)	17 (17.9%)	
AI index	0.48 ± 0.39	0.24 ± 0.24	0.83 ± 0.29	<0.001

### Performance of the CNN Model

**Figure 2:**
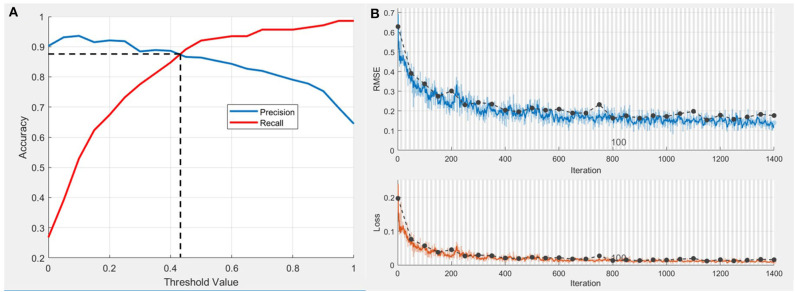
Performance of CNN model in postoperative ONFH prediction. **(A)** Precision-recall curve of test set. The threshold value at Break-Even point is 0.425 and the accuracy at this threshold set is 0.873. **(B)** The change of root mean square error (RMSE) and loss during the training process. Dotted line, RMSE and loss of the training set. Blue wave, RMSE of the validation set. Red wave, loss of the validation set.

A CNN model was established for the extraction of radiograph variables. The precision-recall curve of the test set is shown in Figure 2A, while the threshold value at the break-even point was 0.425. This point was set as the highest sum of sensitivity and specificity. Training accuracy values at this threshold for the training set was 0.903 and 0.873 for the test set. The change in RMSE and loss during the training process are shown in Figure 2B. Deviation of the RMSE in the training set and test set gradually decreased and the two curves leveled off (upper diagram) along with the increase of iterations. Similarly, as the number of iterations increased the deviation in loss between the training set and test set gradually decreased.

### Performance of the Predictive Radiograph AI Variables

**Figure 3:**
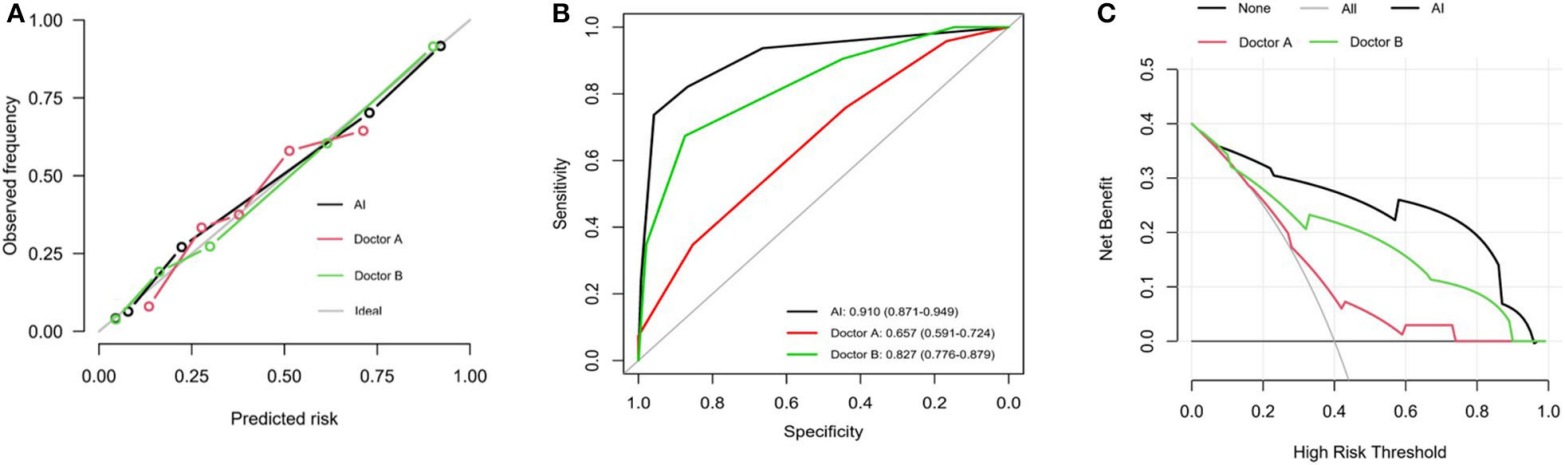
Performance of predictive value of AI index. **(A)** Calibration plots for prediction of AI, Doctor A and Doctor B. Calibration curves depict the calibration of the nomogram in terms of agreement between the predicted risk and outcomes. The 45° gray ideal line represents a perfect Prediction. The closer the dotted line fit is to the ideal line, the better the predictive accuracy of the diagnosis and nomogram is. **(B)** ROC curves for prediction of AI, Doctor A and Doctor B. **(C)** DCA analysis curves for radiodiagnosis of AI, Doctor A and Doctor B. It showed that if the threshold probability is between 0.09 and 0.96, then using the AI index adds more benefit than testing either all or no patients.

The calibration curve of the AI index for the prediction of postoperative ONFH demonstrated good agreement between prediction and actual observations, compared with that of Doctor A and Doctor B (Figure 3A). The sensitivity value was 0.910 (95% CI, 0.871–0.949) for the AI index, 0.657 (95% CI, 0.591–0.724) for the less experienced Doctor A and 0.827 (95% CI, 0.776–0.879) for experienced Doctor B (Figure 3B). The DCA curves shown in Figure 3C indicate that when the threshold probability for a doctor or a patient was within the range of 0.09–0.96, the AI index added more net benefits for the prediction, than that of Doctor A or Doctor B.

### Development of a Hybrid Prediction Model

**Figure 4:**
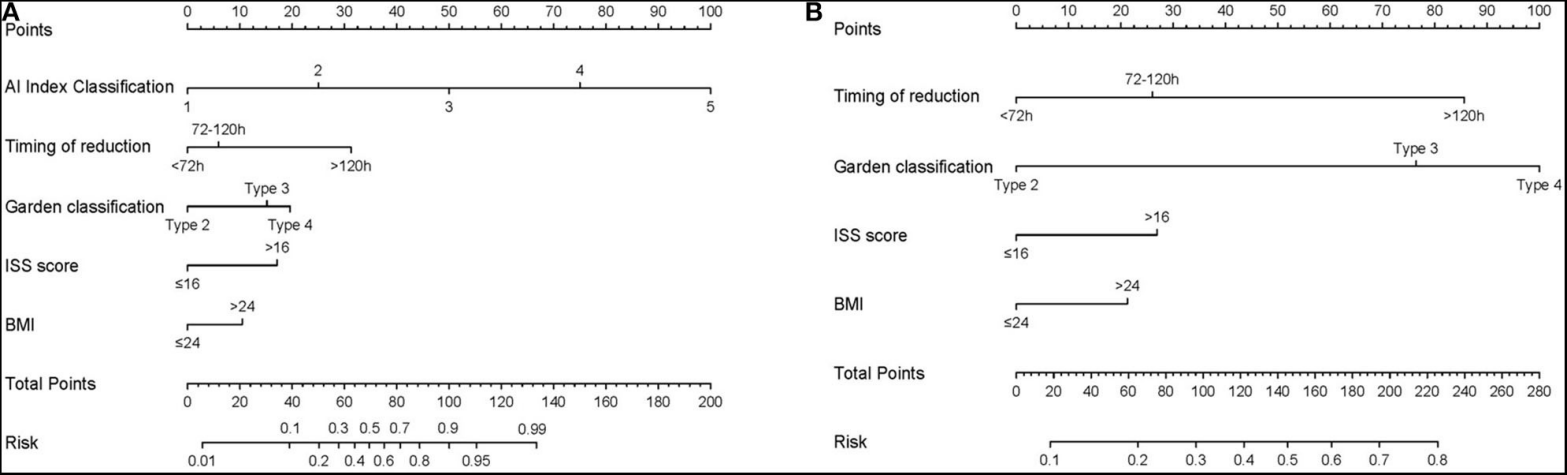
The nomogram for the operative prediction of ONFH. **(A)** Hybrid AI-based nomogram incorporated hybrid independent radiograph and patient variables. **(B)** Clinical-based nomogram constructed based on independent predictors excluded AI index.

In the univariate logistic regression analysis, BMI, Injury Severity Score (ISS), timing of reduction, Garden classification and AI index were found to be significant factors associated with ONFH in the training cohort (all *P* < 0.05; Table 2). In the final multivariate logistic regression model, BMI (HR 0.471, 95% CI 0.187–1.147, *P* = 0.101), ISS (HR 3.427, 95% CI 0.919–13.05, *P* = 0.068), timing of reduction (72 h-120 h: HR 1.533, 95% CI 0.564–4.253, *P* = 0.403; >120 h: HR 9.464, 95% CI 2.471–40.38, *P* = 0.002), Garden classification (Type 3: HR 0.336, 95% CI 0.050–3.315, *P* = 0.292; Type 4: HR 1.344, 95% CI0.243–12.98, *P* = 0.745) and AI index (HR 6.043, 95% CI 4.071–9.717, *P* < 0.001) were identified as hybrid independent predictors of ONFH (Table 3). We then created a prediction nomogram that incorporated the above independent predictors and presented it as a hybrid nomogram (Figure 4A). A clinical nomogram was also constructed based on independent predictors excluded from the AI index (Figure 4B).

**Table 3:** The results of univariate and step-wise multivariate analyses of confounding variables.

**Variable**	**Univariate model**		**Multivariate model**	
	**HR (95% CI)**	***P***	**HR (95% CI)**	***P***
Age	1.013 (0.992–1.035)	0.227	–	–
Sex, male	0.668 (0.395–1.126)	0.131	–	–
BMI, ≤ 24	0.618 (0.361–1.054)	0.077	0.471 (0.187–1.147)	0.101
Smoking, yes	1.083 (0.633–1.847)	0.769	–	–
Alcoholism, yes	1.164 (0.663–2.036)	0.593	–	–
Causes of injury	1.431 (0.851–2.419)	0.147	–	–
ASA grade, grade 2-3	1.412 (0.851–2.419)	0.178	–	–
WIC	1.348 (1.116–1.643)	0.002	Not selected	–
CVD, yes	2.544 (0.964–7.155)	0.063	Not selected	–
ISS score, >16	2.615 (1.178–6.028)	0.020	3.427 (0.919–13.05)	0.068
WBC	0.962 (0.862–1.071)	0.488	–	–
RBC	0.897 (0.567–1.414)	0.640	–	–
PLT	1.002 (0.998–1.007)	0.379	–	–
Hb	1.001 (0.986–1.018)	0.871	–	–
Alb	0.962 (0.885–1.044)	0.358	–	–
D2D	1.411 (0.839–2.382)	0.195	–	–
Timing of reduction			–	–
<72 h	Reference			
72–120 h	1.729 (0.956–3.159)	0.072	1.533 (0.564–4.253)	0.403
>120 h	5.538 (2.562–12.53)	<0.001	9.464 (2.471–40.38)	0.002
Garden classification				
Type 2	Reference		Reference	
Type 3	5.397 (1.443–35.20)	0.029	0.336 (0.050–3.315)	0.292
Type 4	7.150 (1.932–46.41)	0.011	1.344 (0.243–12.98)	0.745
Pauwells angle	0.992 (0.974–1.009)	0.355	–	–
Garden index			Not selected	–
1	Reference		–	–
2	1.645 (0.736–3.774)	0.231	–	–
3	1.956 (0.896–4.400)	0.097	–	–
4	0.942 (0.412–2.181)	0.887	–	–
Interval to part weightbearing			–	–
<1 m	Reference		–	–
1–3 m	0.891 (0.301–2.831)	0.837	–	–
3–6 m	1.368 (0.477–4.241)	0.567	–	–
>6 m	0.625 (0.105–3.207)	0.581	–	–
Interval to full weightbearing			–	–
<3 m	Reference		–	–
3–6 m	1.098 (0.469–2.662)	0.833	–	–
>6 m	0.728 (0.271–1.987)	0.529	–	–
AI index (per 0.25 increase)	4.594 (3.365–6.572)	<0.001	6.043 (4.071–9.717)	<0.001

### Performance of the Hybrid Nomogram

**Figure 5:**
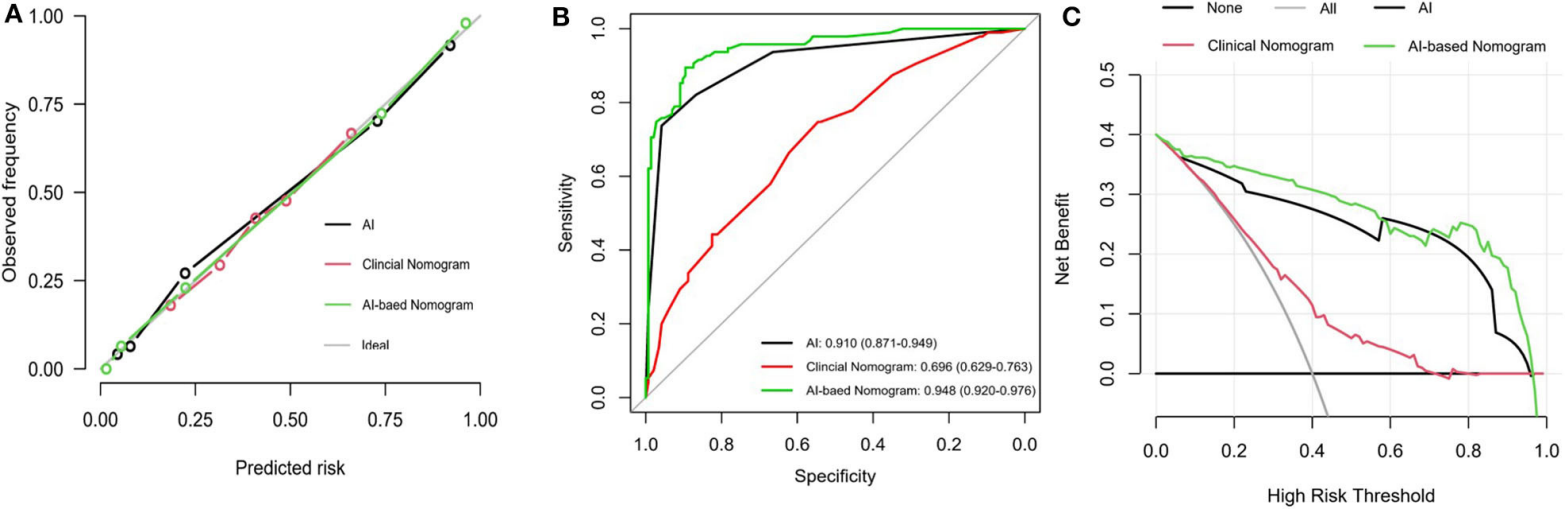
Performance of the hybrid predictive model. **(A)** Calibration plots for AI index, AI-based nomogram and Clinical nomogram. **(B)** ROC curves for prediction of AI index, AI-based nomogram and Clinical nomogram. **(C)** DCA analysis curves for AI radiodiagnosis, AI-based nomogram and Clinical nomogram. The y-axis measures the net benefit. The blue line represents the hybrid AI-based nomogram. The green line represents the clinical nomogram. The gray line represents the assumption that all patients have postoperative ONFH. Thin black line represents the assumption that no patients have postoperative ONFH. The x-axis represents the threshold probability. The threshold probability is where the expected benefit of treatment is equal to the expected benefit of avoiding treatment. It showed that if the threshold probability is between 0 and 0.98, then using the AI-based nomogram adds more benefit in predicting ONFH than testing either all or no patients.

The calibration curve of the hybrid nomogram for the prediction of postoperative ONFH demonstrated good agreement between prediction and actual observations, compared with that of the clinical nomogram (Figure 5A). The AUC of the AI-based nomogram was 0.948 (95% CI, 0.920–0.976), while the AUC for the clinical nomogram was 0.696 (95% CI, 0.629–0.763) (Figure 5B). The difference was statistically significant, which indicated that the hybrid nomogram showed better discrimination and prediction ability for the diagnosis of ONFH.

### Clinical Use

The DCA for the hybrid nomogram and for the clinical nomogram are presented in Figure 5C. The DCA indicated that when the threshold probability for a doctor or a patient was within the range of 0–0.98, the hybrid nomogram added more net benefits than “treat all” or “treat none” strategies. The range for the clinical nomogram was from 0.2 to 0.7, revealing that use of the hybrid nomogram to predict postoperative ONFH was more beneficial.

## Discussion

Early detection and identification of ONFH after femoral neck fracture fixation has been a long-term concern in clinical practice. In this study, we developed and trained a DL model that could use postoperative pelvic radiographs to predict ONFH. The output values of the CNN model successfully stratified patients based on their risk of developing postoperative ONFH, which was referred to as AI index classification for prediction. The predictive performance of the AI index was significantly superior to the predictive performance of a less experienced orthopedic doctor and non-inferior to that of an experienced orthopedic doctor. A combination of patient and radiograph variables were used to construct an AI-based nomogram for postoperative ONFH prediction. The hybrid nomogram showed better performance for the postoperative prediction of ONFH than a single clinical nomogram, indicating its potential in predicting and targeting ONFH during clinical follow-up to provide a decision base for orthopedic doctors.

Hip pain is the most common postoperative symptom after FNF surgery. It may be associated with fractures, surgery, implant irritation, and early ONFH that should be identified during follow-up. Postoperative X-rays are the most common and readily available imaging examination used for routine clinical follow-up after internal fixation. The detection of sclerotic abnormalities and trabecular interruptions of the femoral head for the diagnosis of postoperative ONFH are subjective and depend on the level of experience and diagnostic criteria used by each doctor. Only radiologists who are rich in experience, may be able to accurately predict ONFH using postoperative X-rays. Even then, objectivity and consistency may be difficult to be achieved. The increased workload of radiologists worldwide has already had a significant impact on the diagnostic performance of radiologists (29, 30). Therefore, DL can be used as a potential auxiliary diagnostic tool for orthopedic diagnoses to obtain stable and accurate diagnoses (16, 31). In this study, we trained a DL model to read postoperative X-rays to predict ONFH. The accuracy and consistency of the DL model was significantly better than that of an orthopedic doctor with less experience. The DL model was similar in accuracy but better in consistency, compared with the experienced orthopedic doctor. This indicated the potential of the use of the DL model for the diagnosis and prediction of postoperative ONFH. Previous studies have indicated that an important feature of the DL model is its ability to detect key features of images through cyclic learning undergone by neural networks, which may be different from the existing understanding and research on image features in black box models. This makes it possible for the diagnostic path of the DL model to differ from existing known diagnostic and prediction criteria, resulting in a positive difference in the diagnostic accuracy of the DL model, compared with that of orthopedic doctors. The DL model created in Chee's study showed a high level of sensitivity and accuracy for the diagnosis of pre-collapse ONFH (22). When we applied the CNN network obtained from this non-traumatic ONFH prediction model to our postoperative ONFH prediction, internal fixation of the postoperative X-ray was found to be one of the major differences between the two models. Recent studies have suggested that different fixation constructs, such as cannulated screws or dynamic hip screws, produce different fracture fixation outcomes. The location differences under the implemented operations standard for the same fixation construct do not significantly affect outcomes (32). During training, we found that the output of the DL model could still reflect prediction efficiency and showed good calibration, even though the positions of the metal internal fixations were not exactly the same and occupied the recognition area in the finite image pixel.

Existing studies using clinical risk factors, such as demographic data, fracture classification, and preoperative interval, to make preoperative predictions for surgical decisions (33–35). Due to the lack of the incorporation of all perioperative variables, especially the intraoperative and postoperative radiograph variables, the preoperative prediction models in these studies have shown difficulties in achieving an ideal predictive ability. For example, the clinical nomogram constructed in our study achieved an AUC of 0.696 (95% CI, 0.629–0.763), which is similar to the AUC of 0.746 obtain by the Naive Bayes Classifier constructed by Cui et al. (36). The predictive ability of a preoperative model is limited for patients who have received certain internal fixation, for example dynamic hip screws and cannulated compression screws (34, 36). The hybrid nomogram showed better prediction performance after the incorporation of patient and radiograph variables, compared with conventional clinical nomograms and the simple radiographic-based DL model for postoperative ONFH prediction. In this study, the hybrid classifier achieved an AUC of 0.948 (95% CI, 0.920–0.976). The variables we included after multivariate regression analysis of all risk factors were similar to that of conventional preoperative clinical prediction models. High-risk factors generally include fracture patterns, preoperative interval, and BMI. Inclusion of the DL model-based imaging prediction significantly improved the ONFH predictive ability of the traditional prediction models, indicating the value of using a combination of variables. The predictive model using hybrid variables more closely mimicked the diagnostic and predictive processes of orthopedic doctors, who are better at interpreting images based on the clinical status of patients (37). The addition of a combination of patient and hospital process variables associated with routine clinical care improved the ability of a DL model trained by Badgeley et al. to predict hip fractures (38). One explanation for this improvement was the presence of non-biological signals on radiographs that are predictive of diseases (39). Although multiple regression analyses were performed for risk factors, including intraoperative reduction, and postoperative weight bearing, the variables included in the single clinical nomogram were all preoperative variables. Among them, Garden classification showed the most assigned value, which was similar to the results of previous studies that found that fracture patterns are crucial for the prediction of postoperative ONFH (7, 40). When the postoperative AI index was included, the attribution of Garden classification decreased significantly, which may be because the AI index already included certain manually incorporated graded variables from the images. The information was considered as a non-biological signal and contributed to the classification. The DL-based prediction model that incorporated a combination of patient and radiograph variables showed a significantly higher ability of prediction postoperative ONFH, and can be used to provide second opinions and a base for doctors to make decisions during clinical follow-up.

In the DCA curves analysis, prediction and diagnosis based on the DL model were found to be non-inferior to that of the two orthopedic doctors, while that of the AI-based nomogram using hybrid variables was superior to imaging prediction alone, allowing for more accurate diagnosis and prediction during clinical follow-up. There is no doubt that the gold standard imaging modality for the preliminary stages of ONFH is MRI (41, 42). However, MRI is not the most common test used to evaluate treatment options and ONFH during postoperative FNF follow-up. MRIs are affected by metal implants, which may cause potential internal fixation losses and thermal effect (43). MRI tests are more expensive, take longer, and require the radiologist to have a higher level of diagnostic experience. Nomograms based on the DL model and clinical variables can improve the ability of positive diagnostic screening and provide doctors the opportunity of obtaining a second opinion.

The AI-based nomogram using hybrid variables may potentially assist in decision making during clinical follow-up as patients with early-stage ONFH may benefit from timely interventions (44). Although the definitive method of treatment for traumatic ONFH remains controversial, certain early interventions have been widely used during post-operative clinical follow-up. For patients with a high probability of developing ONFH, interventions for hip preservation or delayed joint replacement, including platelet-rich plasma (PRP)-incorporated autologous granular and free vascularized fibular, have been proven to be safe and effective procedures for postoperative ONFH (45, 46). Extracorporeal shock wave therapy and alendronate administration can also be potentially performed on patients with a moderate probability of a risk of developing ONFH (47–49). We assessed whether the AI-based nomogram assisted decisions that would improve patient outcomes to justify its clinical usefulness. Our study showed that if the threshold probability was between 0.06 and 0.96, as shown by the constructed decision curves, the AI-based nomogram could predict postoperative ONFH compared with treating either all or no patients. This indicated that early postoperative prediction using this hybrid of patient and radiograph variables can be useful for the application of early interventions that may even allow for a reasonable delay of the onset of arthroplasty (50). Substantial positive rehabilitation can be applied after accurate predictions are obtained after the operation for patients with a lower prediction probability, which will also relieve patient anxiety (51).

This study has some limitations. First, it was conducted on a retrospective cohort study, and is therefore likely to have been affected by selection bias. Second, due to the rarity of the disease, our study included only 238 images in the CNN model. The performance of the CNN model can be improved by using a larger multicenter sample size. Third, our diagnostic criteria for postoperative ONFH was based on follow-up MRIs and typical pelvic radiographs without the use of histopathological confirmation. Therefore, false-negative and false-positive values would not have been avoided due to the subjectivity of the imaging diagnosis method. At the same time, transverse comparison was not conducted with gold standard MRI when postoperative X-rays were included 6 months after surgery. The reason was that, as a retrospective study, MRIs had been performed on only 197 patients, probably due to their high cost. In the future, prospective clinical studies using larger cohorts should be preplanned to investigate strategies that can be used for ONFH prediction of patients after internal fixation.

## Conclusion

In conclusion, this study presents a DL facilitated nomogram that incorporates hybrid radiograph and patient variables, shows favorable predictive accuracy for preoperative osteonecrosis of femoral head in patients with femoral neck fractures after internal fixation.

## Data Availability Statement

The raw data supporting the conclusions of this article will be made available by the authors, without undue reservation.

## Ethics Statement

The studies involving human participants were reviewed and approved by the First Affiliated Hospital of USTC. The patients/participants provided their written informed consent to participate in this study.

## Author Contributions

WZ and XZ conceived and designed the study, and wrote the manuscript. WZ collected the data. CZ, BW, and SF read, corrected, and approved the final manuscript. All authors read and approved the final manuscript.

## Conflict of Interest

The authors declare that the research was conducted in the absence of any commercial or financial relationships that could be construed as a potential conflict of interest.
